# The progression rate of spinocerebellar ataxia type 3 varies with disease stage

**DOI:** 10.1186/s12967-022-03428-1

**Published:** 2022-05-14

**Authors:** Linliu Peng, Yun Peng, Zhao Chen, Chunrong Wang, Zhe Long, Huirong Peng, Yuting Shi, Lu Shen, Kun Xia, Vanessa B. Leotti, Laura Bannach Jardim, Beisha Tang, Rong Qiu, Hong Jiang

**Affiliations:** 1 Department of Neurology, Xiangya Hospital, Central South University, Changsha, 410008 Hunan China; 2 School of Basic Medical Science, Central South University, Changsha, 410008 Hunan China; 3 National Clinical Research Center for Geriatric Disorders, Xiangya Hospital, Central South University, Changsha, 410008 Hunan China; 4 Key Laboratory of Hunan Province in Neurodegenerative Disorders, Central South University, Changsha, 410008 Hunan China; 5 Hunan International Scientific and Technological Cooperation Base of Neurodegenerative and Neurogenetic Diseases, Changsha, China; 6 School of Computer Science and Engineering, Central South University, Changsha, 410083 Hunan China; 7 Department of Pathology, Xiangya Hospital, Central South University, Changsha, 410008 Hunan China; 8 Department of Neurology, The Second Xiangya Hospital, Central South University, Changsha, 410011 Hunan China; 9 Center for Medical Genetics, School of Life Sciences, Central South University, Changsha, 410008 Hunan China; 10 Hunan Key Laboratory of Medical Genetics, Central South University, Changsha, 410008 Hunan China; 11 Departamento de Estatística, Universidade Federal do Rio Grande do Sul, Av. Bento Gonçalves, 9500, Porto Alegre, 91509-900 Brazil; 12 Programa de Pós-Graduação em Epidemiologia, Universidade Federal do Rio Grande do Sul, Rua Ramiro Barcelos 2400, Porto Alegre, 90035-003 Brazil; 13 Programa de Pós-Graduação em Ciências Médicas, Universidade Federal do Rio Grande do Sul, Rua Ramiro Barcelos 2400, Porto Alegre, 90035-903 Brazil; 14 Departamento de Medicina Interna, Universidade Federal do Rio Grande do Sul, Rua Ramiro Barcelos 2400, Porto Alegre, 90035-903 Brazil; 15 Medical Genetics Service, Hospital de Clínicas de Porto Alegre, Rua Ramiro Barcelos 2350, Porto Alegre, 90035-903 Brazil

**Keywords:** Spinocerebellar ataxia type 3, CAG repeats, Progression prediction, Growth model

## Abstract

**Background:**

In polyglutamine (polyQ) diseases, the identification of modifiers and the construction of prediction model for progression facilitate genetic counseling, clinical management and therapeutic interventions.

**Methods:**

Data were derived from the longest longitudinal study, with 642 examinations by International Cooperative Ataxia Rating Scale (ICARS) from 82 SCA3 participants. Using different time scales of disease duration, we performed multiple different linear, quadratic and piece-wise linear growth models to fit the relationship between ICARS scores and duration. Models comparison was employed to determine the best-fitting model according to goodness-of-fit tests, and the analysis of variance among nested models.

**Results:**

An acceleration was detected after 13 years of duration: ICARS scores progressed 2.445 (SE: 0.185) points/year before and 3.547 (SE: 0.312) points/year after this deadline. Piece-wise growth model fitted better to studied data than other two types of models. The length of expanded CAG repeat (CAGexp) in *ATXN3* gene significantly influenced progression. Age at onset of gait ataxia (AOga), a proxy for aging process, was not an independent modifier but affected the correlation between CAGexp and progression. Additionally, gender had no significant effect on progression rate of ICARS. The piece-wise growth models were determined as the predictive models, and ICARS predictions from related models were available.

**Conclusions:**

We first confirmed that ICARS progressed as a nonlinear pattern and varied according to different stages in SCA3. In addition to *ATXN3* CAGexp, AOga or aging process regulated the progression by interacting with CAGexp.

**Supplementary Information:**

The online version contains supplementary material available at 10.1186/s12967-022-03428-1.

## Introduction

Spinocerebellar ataxia 3 (SCA3), an autosomal dominant neurodegenerative disorder, is widely regarded as the most common subtype of SCA worldwide. It also belongs to the group of polyglutamine (polyQ) diseases, which includes ten inherited neurodegenerative diseases such as Huntington’s disease (HD) [1–3]. SCA3 arises from an expanded CAG repeat (CAGexp) in *ATXN3* gene, which is hallmarked by progressive cerebellar ataxia and variable features including pyramidal syndrome, extrapyramidal signs as well as peripheral neuropathy [4, 5].

There is an unmet clinical need for further characterization of long-term disease progression and natural history. Meanwhile, further identification of underlying factors and the construction of prediction models for progression are also of great value. This would contribute to genetic counseling, clinical management and therapeutic interventions [6–8].

Time scales could influence the result of disease progression or outcome occurrence and even lead to contradictory findings [9–11]. Furthermore, substantial evidence indicated that the trajectories of disease progression displayed a non-linear change in various polyQ diseases, including HD, SCA2, and SCA6, etc. [12–17]. Recently, Leotti et al. performed the longest longitudinal study in a Dutch SCA3 cohort of 82 participants over a period of 15 years. Using linear growth curve model, they only assessed the linear pattern of disease progression as measured by the International Cooperative Ataxia Rating Scale (ICARS) according to the time scale of age [18].

In our present study, using multiple different linear and non-linear models with a different time scale of disease duration, we reanalyzed the previously published data of Leotti et al. for the following purposes: (1) to assess the potential impact of different time scales on study results; (2) to investigate whether ICARS progression in SCA3 follows a non-linear pattern according to disease duration; (3) to explore whether the speed of progression differed across early or late stages of the disease duration; (4) to develop an appropriate model to predict disease progression of SCA3 after considering non-linear effects.

## Materials and methods

### Subjects

Clinical data from 82 symptomatic SCA3 patients were derived from an earlier report by Leotti et al. [18]. In this study, all SCA3 subjects were followed up in the University of Groningen Medical Center, Netherlands, between 2002 and 2017. Disease progression of patients was measured by ICARS and 642 complete ICARS examinations were publicly available. This report did not need the approval of the local Ethics. More detailed information has been reported previously, and will not be covered in this paper [18].

### Statistical analysis

We removed one outlier sample with disease duration of more than 40 years considering its influence on statistical results. Finally, 81 participants with 634 complete ICARS examinations were included for our analysis. Disease duration was defined as the interval between the patient’s age of examination and AOga as previously reported by Leotti et al. [18]. In contrast to this study, we chose disease duration as the timescale, rather than the age of examination.

Linear growth models were first employed to model the linear relationship between ICARS scores and duration for each patient. Further, a quadratic growth model was conducted to explore whether the trajectory of progression tended to a non-linear pattern. Moreover, we used piece-wise (two-segment in our analysis) linear growth models to investigate whether progression rates varied in the early or late stages. The piece-wise growth curve model is a flexible approach to model the nonlinear growth form. It splits the curvilinear growth trend into separate linear segments connected by inflection points or knots, thus capturing key features of change for each segment [19, 20].

In this study, dt was regarded as the total duration of SCA3 to fit linear or quadratic growth models. Similar to one previous study [21], to fit two-segment linear growth models at each inflection point (I) of duration, we defined two new variables (d1 and d2): d1 = dt − I for dt ≤ I but d1 = 0 for dt > I; and d2 = 0 for dt ≤ I but d2 = dt − I for dt > I. Here, d1 indicated an early stage whereas d2 represented a late stage.

We calculated the progression rates on the annual scores of ICARS. The effects of CAGexp, AOga and gender on the progression of ICARS were also analyzed. Such three potential factors were included in the models as fixed effects. Obviously, in our analysis, AOga could also serve as a proxy for the age at the time of examination to investigate the aging process on clinical progression, as described by other similar studies in HD [22, 23]. Our analytical data were collected naturalistically at irregular intervals from SCA3 subjects who entered follow-up at various stages. Therefore, age at first assessment has little meaning as a proxy for age during follow-up [24]. Moreover, this study applied an unstructured covariance matrix for random intercepts and slopes effects to explain the heterogeneity of individuals regarding baseline measurement and trajectory.

All data analysis and mapping were undertaken in R software (version 3.5.3). Two-tailed tests were used and *P* < 0.05 indicated statistical significance.

### Models evaluation

The goodness-of-fit statistics tests were performed based on a set of evaluation criteria, including Akaike’s information criterion (AIC), Bayesian information criterion (BIC), log-likelihood (logLike), and Nakagawa R-squared (Nakagawa R^2^, containing marginal and conditional R^2^) [25, 26]. Also, we conducted the analysis of variance (ANOVA) through likelihood ratio tests to compare the fits of different models.

Models comparison was employed to select the best-fitting model according to the goodness-of-fit tests and ANOVA analysis results. In general, the best-fitted model has the smallest values of AIC, and BIC, along with the highest logLike and Nakagawa R^2^ value [25–28]. If p-value in ANOVA is less than 0.05, related models differ significantly [29–31].

## Results

### Description of the participants

After omitting the outlier sample, the general characteristics of the final analysis cohort showed that 81 participants (43 for women) were followed up for 8.280 (SD: 4.190) years on average. The average CAGexp repeat length at *ATXN3* was 67.700 (SD: 3.650). And the mean duration at the first examination was 7.110 (SD: 5.030) years, along with the mean AOga of 42.100 (SD: 9.880) years (Table 1). The initial characteristics of the study cohort were provided in the previous article by Leotti et al., which were not described in detail here [18].

**Table 1:** General characteristics of the final analysis data after the removal of an outlier sample

	Patients
Total subjects, No	81
Gender (female), No. (%)	43(53.09%)
Mean number of examinations per participant, Mean ± SD [Range]	7.830 ± 3.670 [1–16]
Mean number of years of follow up per participant, Mean ± SD [Range]	8.280 ± 4.190 [0–15.300]
Mean interval between visits per participant (years), Mean ± SD [Range]	1.210 ± 0.783 [0.071–7.010]
*ATXN3* CAGexp, Mean ± SD [Range]	67.700 ± 3.650 [60.000–75.000]
AOga (years), Mean ± SD [Range]	42.100 ± 9.880 [20.000–72.000]
Disease duration at first examination (years), Mean ± SD [Range]	7.110 ± 5.030 [0.529–24.500]
Age at first examination (years), Mean ± SD [Range]	49.200 ± 11.300 [22.200–77.000]
ICARS score at first examination, Mean ± SD [Range]	21.600 ± 15.500 [2.000–81.000]

No. = Number; SD = standard deviation; *ATXN3* CAGexp = the length of expanded *ATXN3* allele; AOga = Age at onset of gait ataxia

### The estimated parameters of different growth models

**Fig. 1:**
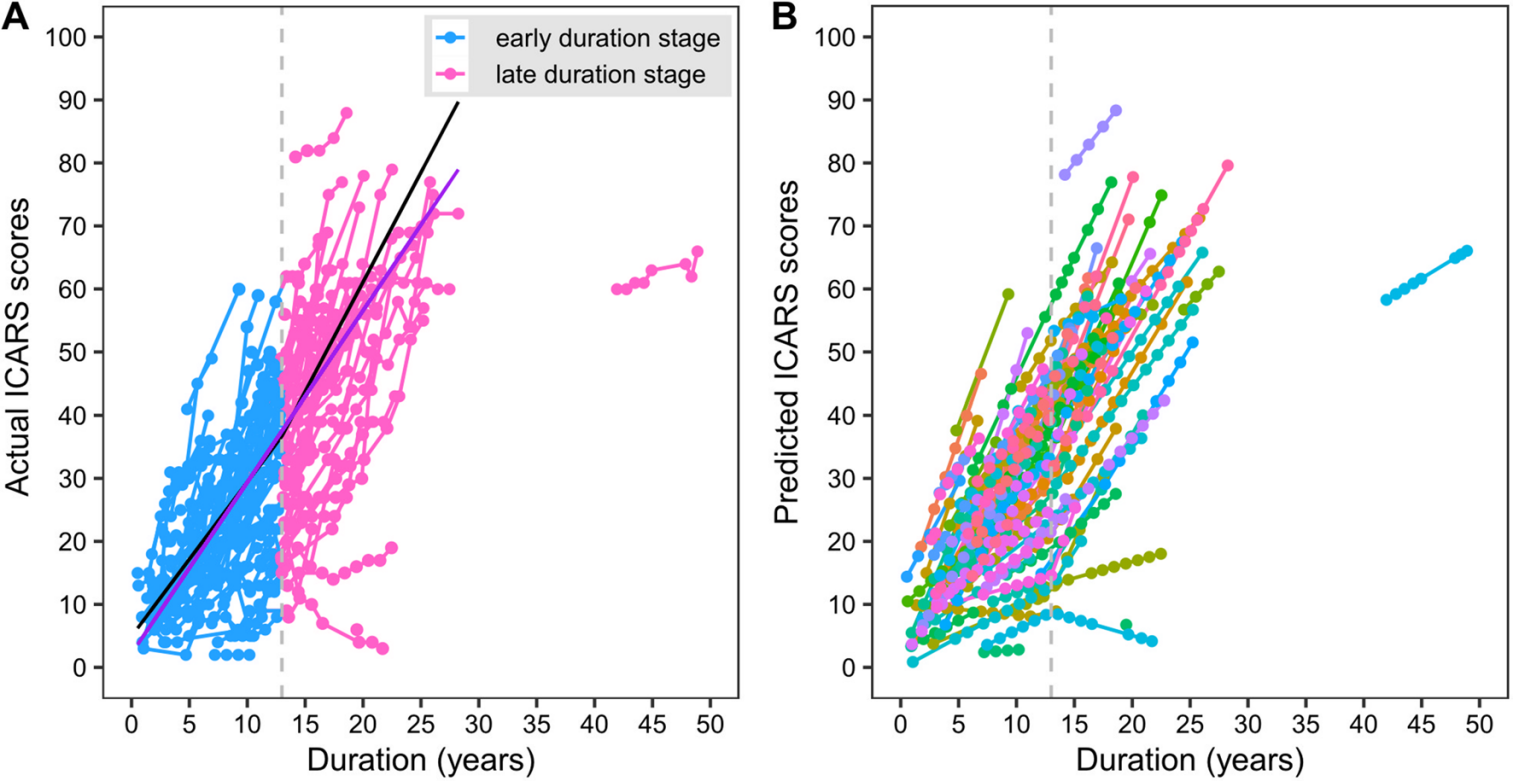
The trajectory and average progression rates of ICARS over the studied period. **A** showed the actual trajectory of ICARS for each participant. The purple line depicted the average progression rate from LM1 model with single-slope, with black lines showing the average progression rate from PM1 model with two-segment slopes. Dots were plotted for each subject indicating actual ICARS scores (y-axis) versus disease duration (x-axis). The blue dots and lines represented early disease stage, with red dots and lines for late stage. **B** showed the predicted trajectory of ICARS for each participant using the optimal PM4c growth model. Different colored dots and lines represented predicted ICARS scores (y-axis) versus disease duration (x-axis) for different subjects

The ICARS scores of each subject over the period were shown in Fig. 1A. ICARS scores progressed by an average of 2.744 (SE: 0.154) points/year during the total duration in LM1 model (Table 2). We compared the value of logLike, AIC, and BIC to determine the inflection point in the two-segment growth model, with 18 inflection point (at a duration of 3, 5, 7, 9, 10, 11, 12, 13, 14, 15, 16, 17, 18, 19, 20, 25, 30, 35 years) being tested. Finally, it got the best fit at about 13 years of duration in terms of logLike, AIC and BIC (data was not shown).

**Table 2:** Parameter estimates and fit statistics of the fitted models for progression rate of ICARS in SCA3 patients

	LM1	LM2	LM3	LM4	QM1	PM1	PM2	PM2c	PM3	PM3c	PM4	PM4c
Parameter estimates of fixed effects												
Intercept	2.067 (1.309) P = 0.115	44.448 (24.183) P = 0.067	− 16.464 (5.448) P = 0.003	− 60.517 (38.837) P = 0.120	10.726 (1.915) P < 0.001	36.746 (1.955) P < 0.001	− 59.997 (35.214) P = 0.089	− 62.620 (31.899) P = 0.050	35.501 (8.876) P < 0.001	36.472 (8.274) P < 0.001	− 217.508 (57.354) P < 0.001	− 216.416 (49.701) P < 0.001
dt	2.744 (0.154) P < 0.001	− 7.940 (2.670) P = 0.003	4.050 (0.702) P < 0.001	− 12.590 (4.346) P = 0.005	1.199 (0.280) P < 0.001	–	–	–	–	–	–	–
dt^2^	–	–	–	–	0.069 (0.009) P < 0.001	–	–	–	–	–	–	–
d1	–	–	–	–	–	2.445 (0.185) P < 0.001	− 7.264 (3.305) P = 0.028	− 7.582 (2.771) P = 0.006	3.671 (0.817) P < 0.001	3.787 (0.723) P < 0.001	− 9.944 (5.672) P = 0.080	− 9.814 (4.606) P = 0.034
d2	–	–	–	–	–	3.547 (0.312) P < 0.001	− 7.516 (5.989) P = 0.210	− 6.604 (2.785) P = 0.018	5.323 (1.582) P < 0.001	4.878 (0.765) P < 0.001	− 8.414 (9.030) P = 0.352	− 8.745 (4.618) P = 0.059
CAGexp	–	− 0.623 (0.356) P = 0.084	–	0.562 (0.485) P = 0.250	–	–	1.429 (0.519) P = 0.007	− 0.460 (0.328) P = 0.164	–	–	3.195 (0.716) P < 0.001	0.970 (0.449) P = 0.034
dt*CAGexp	–	0.157 (0.039) P < 0.001	–	0.205 (0.054) P < 0.001	–	–	–	0.148 (0.041) P < 0.001	–	–	–	0.171 (0.058) P = 0.003
d1*CAGexp	–	–	–	–	–	–	0.144 (0.049) P = 0.003	–	–	–	0.171 (0.070) P = 0.016	–
d2*CAGexp	–	–	–	–	–	–	0.162 (0.088) P = 0.067	–	–	–	0.172 (0.113) P = 0.128	–
AOga	–	–	0.446 (0.128) P < 0.001	0.590 (0.184) P = 0.002	–	–	–	–	0.026 (0.206) P = 0.898	0.424 (0.112) P < 0.001	0.895 (0.271) P = 0.002	0.680 (0.168) P < 0.001
dt*AOga	–	–	− 0.032 (0.016) P = 0.055	0.025 (0.021) P = 0.232	–	–	–	–	–	− 0.032 (0.017) P = 0.055	–	0.015 (0.022) P = 0.513
d1*AOga	–	–	–	–	–	–	–	–	− 0.030 (0.019) P = 0.123	–	0.018 (0.027) P = 0.517	–
d2*AOga	–	–	–	–	–	–	–	–	− 0.043 (0.037) P = 0.251	–	0.005 (0.047) P = 0.917	–
Fit statistics												
AIC	4019.470	4013.763	4021.004	4005.769	3984.778	3945.904	3947.395	3942.736	3952.541	3946.137	3939.014	3928.450
BIC	4046.163	4049.328	4056.57	4050.194	4015.909	3990.377	4005.148	3996.066	4010.294	3999.467	4010.018	3990.623
logLik	− 2003.735	− 1998.881	− 2002.502	− 1992.884	− 1985.389	− 1962.952	− 1960.697	− 1959.368	− 1963.270	− 1961.069	− 1953.507	− 1950.225
Conditional R^2^	0.970	0.971	0.971	0.972	0.978	0.979	0.979	0.979	0.979	0.979	0.980	0.980
Marginal R^2^	0.479	0.540	0.480	0.623	0.488	0.517	0.575	0.575	0.516	0.517	0.641	0.643
P-value in ANOVA	–	P^a^ = 0.008	P^a^ = 0.292	P^a^ < 0.001	P^a^ < 0.001 P^b^ < 0.001	P^a^ < 0.001	P^a^ = 0.212 P^b^ < 0.001	P^b^ = 0.028 P^c^ < 0.001	P^b^ = 0.888 P^c^ < 0.001	P^b^ = 0.152 P^c^ < 0.001	P^b^ = 0.004 P^c^ < 0.001	P^b^ < 0.001 P^c^ < 0.001

LM1: linear growth model (duration as a variable)

LM2: linear growth model (duration and CAGexp as variables)

LM3: linear growth model (duration and AOga as variables)

LM4: linear growth model (duration, CAGexp and AOga as variables)

QM1: quadratic growth model (duration and duration^2 as variables)

PM1: piece-wise linear growth model (duration as a variable)

PM2: piece-wise linear growth model (duration and CAGexp as variables)

PM2c: piece-wise linear growth model (piece-wise fitting for duration as a variable, linear fitting for CAGexp as a variable)

PM3: piece-wise linear growth model (duration and AOga as variables)

PM3c: piece-wise linear growth model (piece-wise fitting for duration as a variable, linear fitting for AOga as a variable)

PM4: piece-wise linear growth model (duration, CAGexp and AOga as variables)

PM4c: piece-wise linear growth model (piece-wise fitting for duration as a variable, linear fitting for CAGexp and AOga as variables)

Nakagawa R^2^, usually interpreted as pseudo r-squared, which indicates the amount of heterogeneity accounted for by the fitted model. It includes two types of R^2^ (marginal and conditional R^2^). The marginal R^2^ relates to the variance of the fixed effects, while conditional R^2^ takes both the fixed and random effects into account. The p-values in ANOVA for model comparison among linear growth model of LM1 null model and other related models were marked by a in each column (i.e., LM1 vs LM2, LM1 vs LM3, LM1 vs LM4, LM1 vs QM1, LM1 vs PM1, respectively). And the ANOVA results for piece-wise growth model of PM1 null model vs quadratic growth model and other piece-wise models were expressed by b (i.e., PM1 vs QM1, PM1 vs PM2, PM1 vs PM2c, PM1 vs PM3, PM1 vs PM3c, PM1 vs PM4, PM1 vs PM4c, respectively), while related linear growth models vs their respective corresponding piece-wise models were denoted by c (i.e., LM1 vs PM1, LM2 vs PM2, LM2 vs PM2c, LM3 vs PM3, LM3 vs PM3c, LM4 vs PM4, and LM4 vs PM4c, respectively). The value of parameter estimates of fixed effects were represented by mean [SE]

ICARS = International Cooperative Ataxia Rating Scale; SE = standard error; dt = entire duration; d1 = early duration; d2 = late duration; CAGexp = expanded CAG repeat; AOga = age at onset of gait ataxia; AIC = Akaike’s information criterion, BIC = Bayesian information criterion (BIC); logLike = Log-Likelihood; R^2^ = R-squared; ANOVA = analysis of variance

Based on this knot, we fitted the PM1 model (seen Table 2) and found that the average progression rates of ICARS before and after it were 2.445 (SE: 0.185, P < 0.001) and 3.547 (SE: 0.312, P < 0.001) points/year, respectively (Table 2, Fig. 1A). The single-slope of LM1 model and the double-slope of PM1 model differed in progression rate, especially after 13 years of duration (Fig. 1A).

Compared with LM1 and QM1 models, PM1 model showed notable superiority in terms of the goodness-of-fit criteria (LM1, QM1 and PM1 model with logLike of − 2003.735, − 1985.389, − 1962.952; AIC of 4019.470, 3984.778, 3945.904; BIC of 4046.163, 4015.909, 3990.377; conditional R^2^ of 0.970, 0.978 and 0.979, respectively). ANOVA analysis also showed that PM1 model differed significantly from LM1 and QM1 model (both *P* < 0.001). Therefore, the piece-wise growth model fit the studied data better than other two types of models (i.e., linear and quadratic growth model) (Table 2).

LM2 and PM2c models suggested that *ATXN3* CAGexp repeat length significantly influenced the progression rate (both *P* < 0.001 for the slopes of CAGexp). It was further confirmed by the fact that LM2 and PM2c models showed superiority over their corresponding null models (i.e., LM2 vs LM1 model; and PM2c vs PM1 model, respectively). AOga was not a direct modifier of ICARS progression rate, since related *P* values for the slopes of AOga were all greater than 0.05 in three models of LM3, PM3, and PM3c with AOga as a variable. Interestingly, LM4 and PM4c models combining both CAGexp and AOga as co-modifiers displayed significant improvements in evaluation indicators over their counterpart models only including CAGexp (i.e., LM4 vs LM2 model; and PM4c vs PM2c model, respectively). Incorporating AOga increased the effect size of the effect of CAGexp on ICARS progression. According to these results, it can be deduced that AOga, a proxy for age during follow-up, was not an independent modifier of progression rate but affected the correlation between CAGexp and progression.

These models of PM2c, PM3c, and PM4c with single-slope of *ATXN3* CAGexp repeat length or/and AOga, were superior to their respective corresponding models with two-segment slopes of related modifiers, respectively (i.e., PM2c vs PM2, PM3c vs PM3, and PM4c vs PM4, respectively). Furthermore, in the latter models, the fixed slopes of the two studied modifiers in early-stage basically coincided with that in the following late-stage. These results suggested that the difference in ICARS progression rate between the early and late disease stages may not be attributed to CAGexp and AOga.

We also examined the effect of gender on clinical progression. And the results showed that the *P* values associated with the fixed slopes of gender did not reach statistical significance in two models of LM5 and PM5 with gender as a potential influencing factor (i.e., all related *P* values were greater than 0.05) (Additional file 1). These results suggested that gender had no significant effect on ICARS progression.

PM4c model had the lowest value of AIC (3928.450) and BIC (3990.623), as well as the highest value of logLike (− 1950.225) and marginal R^2^ of Nakagawa R^2^ (0.643) in goodness-of-fit tests. In ANOVA analysis, the PM4c model differed significantly from LM4 (P < 0.001) and PM4 models (P = 0.037). Therefore, the PM4c model was the best fit to the studied data according to our model evaluation criteria (Table 2).

### The predictive results of the optimal growth model

**Fig. 2:**
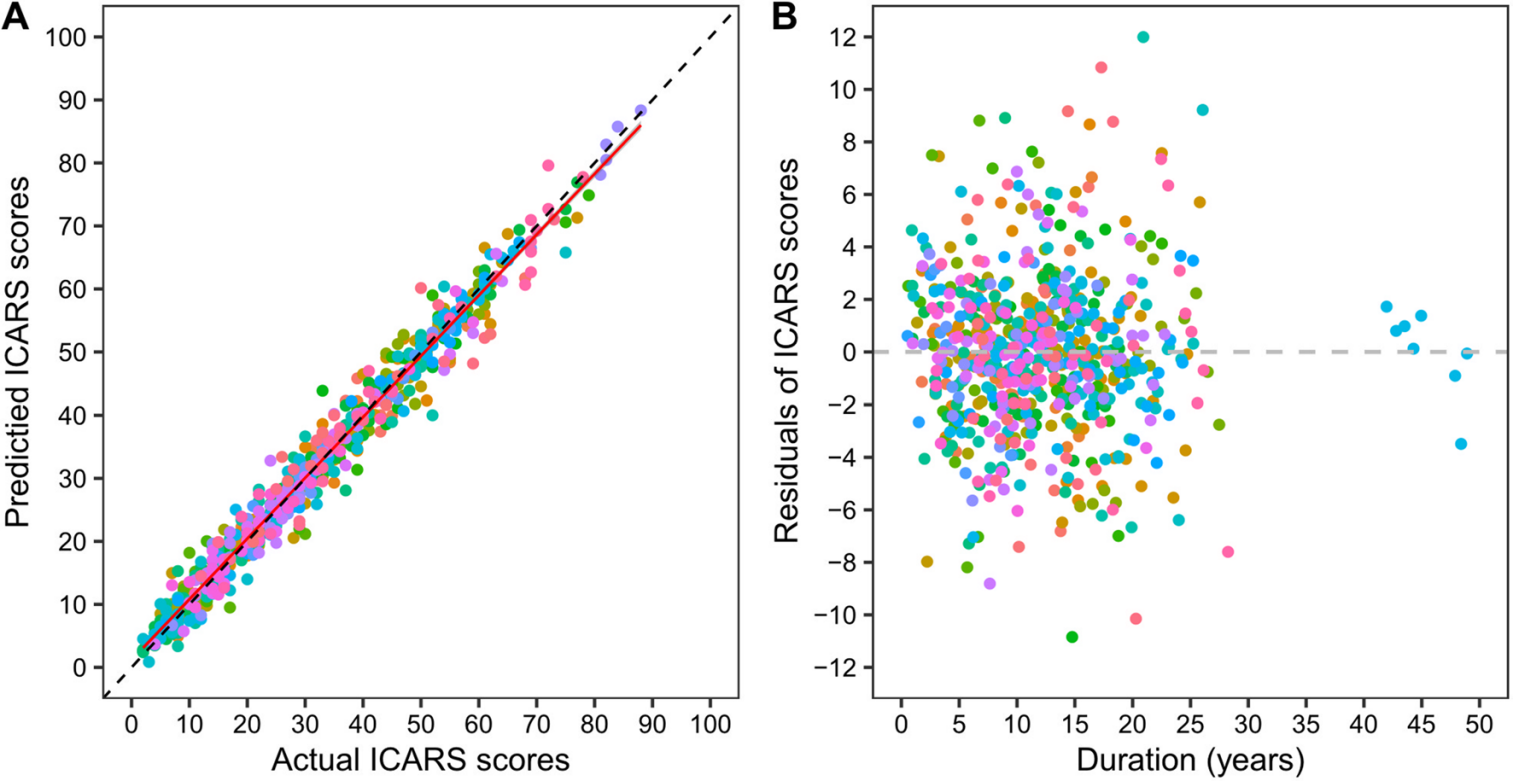
The differences and residuals between the actual and predicted ICARS scores. **A** Displayed the differences between the predicted and actual ICARS scores for all subjects. Different colored dots were used for predicted ICARS scores (y-axis) vs true ICARS scores (x-axis), with a red regression line of optimal fitting of points, and a shadow representing 95% confidence interval of this line. The dotted black line was the ideal line, where true equaled predicted ICARS scores. **B** in this figure showed the residual of ICARS (predicted minus actual values) versus disease duration. The dotted black line was the center line of zero. Different colored dots illustrated the residual (y-axis) versus disease duration (x-axis) for different subjects

Using the optimal PM4c growth model, we obtained the predicted ICARS scores of each participant (Fig. 1B). The general conformity between the predicted and actual trajectory of ICARS progression could be detected by comparing Fig. 1A and B. Also, a scatter graph was used to show the differences between the actual and predicted ICARS scores for all subjects. Most of the ICARS scores predicted by the PM4c model based on CAGexp repeat length of *ATXN3* and AOga were approximate to the actual values of ICARS. Further, its optimal fitted regression line was very close to the ideal line (i.e., predicted equaled actual ICARS) (Fig. 2A).

**Fig. 3:**
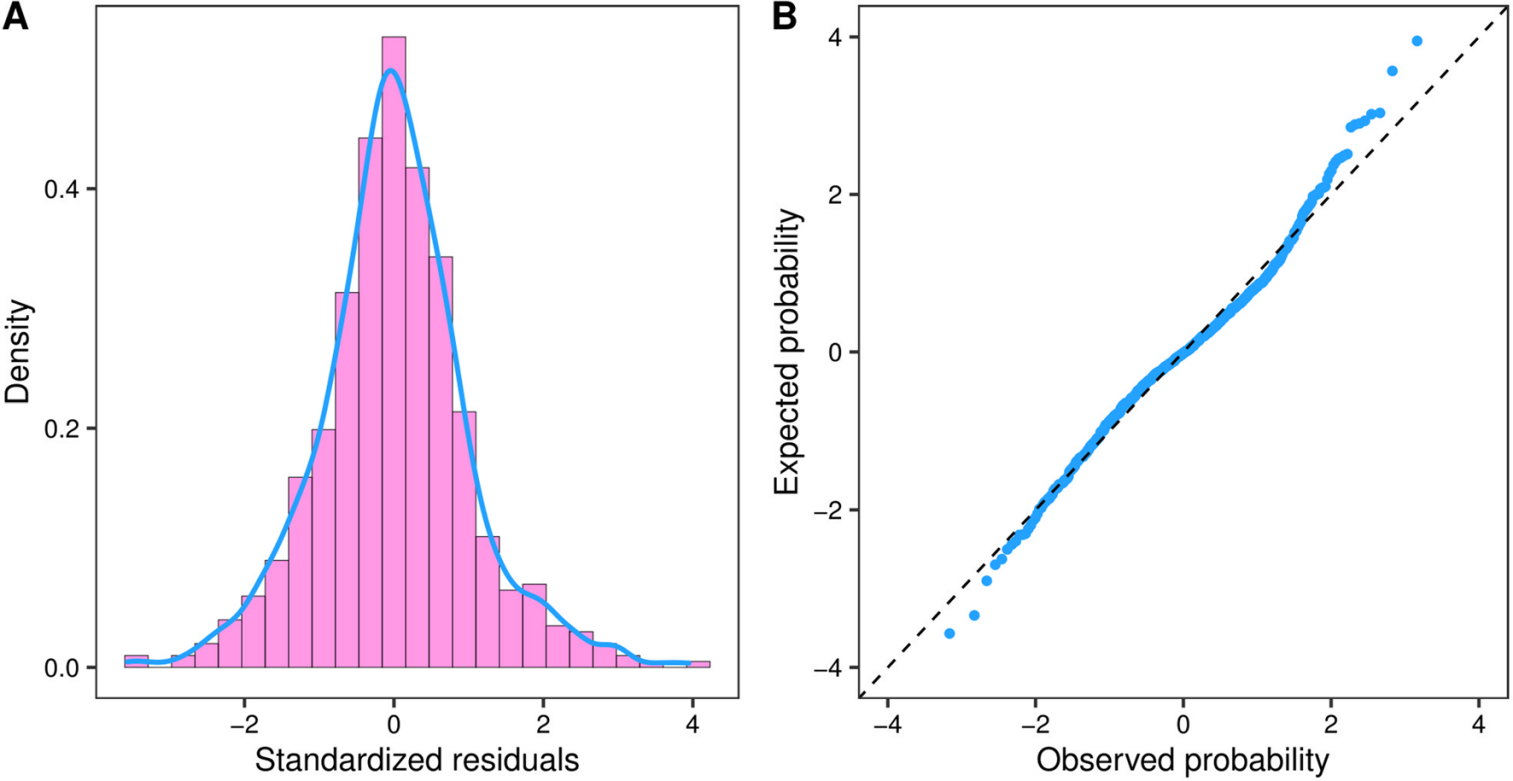
The normality test result of residuals of ICARS scores. **A** was the histogram of ICARS residuals which plotted the distribution of the residuals, with blue curve representing the density curve. **B** was the normal quantile–quantile (Q–Q) plot of ICARS residuals. Blue dots denoted the expected orders of the residuals in a theoretical normal distribution (y-axis) against the observed ordered values of the residuals (y-axis). The adjacent blue dots were connected by blue lines. The dashed black lines were the reference where the expected orders of residuals equal the actual orders of residuals

The residual of ICARS versus duration was equally distributed around the centerline of zero in the residual plot (Fig. 2B). Visually, the residual histogram superimposed with the density function illustrated an approximately normal distribution of residuals (Fig. 3A). The normality assumption was also justified by the normal quantile–quantile (Q–Q) plot because the standardized residuals followed a straight diagonal line (Fig. 3B). These results indicated that the PM4c model was appropriate and fitted well to our current data.

Based on the PM4c model, we provided a excel spreadsheet to predict the average scores and progression of ICARS according to *ATXN3* CAGexp repeat length together with AOga (Additional file 2). In addition, in the case of an unknown AOga, the PM2c model including CAGexp as the only modifier, would be more appropriate for ICARS prediction than the PM4c model. Also, a related spreadsheet was available to the estimate of ICARS (Additional file 3). For example, according to the PM2c model, an individual with 60 CAGexp repeats in *ATXN3* gene at 5 years after AOga would have an average ICARS score of 14.836. Given that his AOga is 55 years, the average ICARS scores at the duration of 5 years predicted by the PM4 model would be 13.121.

### The assessment results of collinearity effect

We calculated the variance inflation factor (VIF) value of AOga against CAGexp to assess the collinearity effect according to the formula of VIF and the AOga variance explained by CAGexp (49% by our study [18] and about 50% in the previous studies [32]). Generally, a VIF value exceeding 5 or 10 suggests a problematic sign of collinearity requiring correction [33, 34]. In this study, related VIF was estimated to be about 2, which was lower than the cut-off level for VIF. Additionally, the fit statistics or estimates of models combining AOga and CAGexp (LM4, PM4, and PM4c model) were basically consistent with their counterpart models with the residual of AOga (RAO) and CAGexp as co-modifiers (LM6, PM6, and PM6c model, respectively) (Additional file 1). Therefore, it is generally reasonable and acceptable to ignore the collinearity between the length of CAGexp and AOga.

## Discussion

Using a different time scale of disease duration, we conducted a new analysis of the ICARS progression rate in the longest longitudinal study of Dutch SCA3 cohort [18]. Compared with the previous age-index prediction models, our duration-index models have some advantages and can yield unique valuable insights into disease progression: (1) it is appropriate to reflect the natural timescale of SCA3 because of the strong correlation between duration and clinical severity or progression [17, 35, 36]; (2) it is more relevant because at baseline the population is constrained to be within the patients having already developed SCA3, while age as time scale can amplify the a priori dis-synchronization of disease process between patients; (3) it may be better suited to be used in future clinical trials since it is a predictor of outcomes and some interventions can be used at different disease stages; (4) it is also useful to investigate whether the rates of ICARS progression changed by duration.

The results showed that using duration as time scale, the piece-wise growth model provided much better fit to the ICARS progression trajectory than linear and quadratic growth model. The progression rates of ICARS varied during the long period of SCA3 patients. Trajectories of ICARS progression were characterized by nonlinear trend and could change with different stages. At early-stage, i.e., in the first 13 years of duration, ICARS progressed more slowly than in the following process after 13 years. Compared to the initial analysis results by Leotti et al. [18], our findings provide novel insights on the progression of SCA3 from another new perspective. In SCA3, this is the first study to suggest the non-linear pattern of disease progression during the long disease duration by comparing multiple linear and nonlinear growth models.

Similar phenomena were also observed in a variety of polyQ diseases including SCA2 [15, 37], SCA6 [17, 38], and HD [12, 39, 40], etc. For example, in SCA2, the disease progression rates, as measured by Scale for the Assessment and Rating of Ataxia (SARA) and Neurological Examination Score for Spinocerebellar Ataxias (NESSCA), were also not uniform during the disease process: early phases of disease duration were related to slower progressions [15]. In SCA6, two studies suggested a similar non-linear pattern of decline on SARA and Inventory of Non-Ataxia Symptoms (INAS) [17, 38]. As for HD, one study showed that the annual growth rate of chorea was greater in the earlier-stage than in the advanced stage [39]. While the total motor score, assessed in the Unified Huntington’s Disease Rating Scale (UHDRS), exhibited a relatively faster rate of progression in patients at mid-stage than those at early and late stage [12]. Our findings, together with these previous reports, suggested that the non-linear progression pattern of clinical scales was plausible and disease progression may not be at the same rate throughout its course.

It might reflect the true biological effect of these diseases. Data from other objective imaging studies were also consistent with this natural phenomenon. For instance, the rate of caudate atrophy was positively correlated with disease duration. It seemed to progress more slowly in pre-symptomatic and early HD patients [41]. The MRI white matter-ventricle scores displayed similar nonlinear trajectories with clinical motor-cognitive scores across the disease span in HD, with a slow, nonlinear progression pattern over time in gray matter loss [40]. Furthermore, the ventricular enlargement rates of HD, which reflect the extension of pathology to extra-striatal gray matter and white matter regions, also accelerated with the prolongation of disease duration [13]. Except for the biological causes, scale limitations may also explain this non-linearity progression pattern of rating scales. Current clinical measures of disease severity and outcome are limited by the floor and ceiling effects and lack sensitivity to early signs and changes over time [12, 15]. On the one hand, in polyQ disease, a large proportion of patients at early-stage have subtle and vague signs but were poorly detected by scales. This might be associated with the slower slope of progression rate in the early stage as shown by our results and other similar findings [15]. On the other hand, disease severity may not coincide with relevant clinical scales. We postulated that the inability of related scales to assess the progression after a certain disease stage may contribute to explain the slowdown of chorea and total motor score in the late stage of HD as mentioned above [12, 39]. Notably, our results showed that CAGexp repeat length and AOga might not be the causes for the different progression rates at different stages. Regardless of the mechanism behind it, the direct use of linear models during prospective longitudinal studies, without consideration of the differences in disease duration, may ignore the non-linear progression pattern.

Consistent with previous results of Leotti et al. [18], we also confirmed that the *ATXN3* CAGexp repeat length significantly influenced the speeding ICARS progression. Unlike this study, we investigated the impact of gender on disease progression. The results indicated that gender was not a significant modifier of ICARS progression. Similarly, there was no significant trend of association between ICARS increase and gender in one previous SCA3 study [42]. Furthermore, three studies found that gender had no significant effect on SARA progression in SCA3, as well as in other any SCA type, including SCA1, SCA2 and SCA6 [6, 17, 43]. Also, gender could not influence the NESSCA progression of SCA3 patients [44]. However, two studies showed that the rate of INAS progression in SCA3 depended on gender, with faster increase in female patients than male patients [6, 17]. According to our results and those previous studies, it can be suggested that there were no consistent conclusions and consensus on whether gender had an effect on disease progression in SCAs patients. Various scales measuring clinical progression among studies might contribute these inconsistent findings. We suspected that gender may not affect the increase of ataxia symptoms measured by ICARS, SARA and NESSCA, but the non-ataxic signs in INAS. Also, different ethnic or population background and observation or follow-up period among these studies may account for the discrepancies.

Additionally, we focused on the impact of AOga rather than RAO on disease progression. It might be more convenient and easier to be popularized clinically, because: (1) a model fitting the relationship between AOga and CAGexp is not required to calculate RAO; (2) accurate prediction of AOga based on CAGexp is still a great challenge. Our results showed that all related *P* values of the slopes for AOga were greater than 0.05 in all models with AOga as the only modifier or combining AOga and CAGexp together. Similar phenomena were also observed in related models combining RAO and CAGexp as co-modifiers. It suggested that AOga (also a proxy for age during follow-up in our study) and RAO could not directly influence the progression rate of ICARS even if combining with CAGexp, which was different from this previous study of Leotti et al. [18].

Similarly, some SCA3 studies also did not found a significant effect of AOga on the progression of ataxia as measured by ICARS and SARA [6, 17]. Whereas two studies demonstrated a correlation between AOga and the non-ataxia progression rate, which was assessed by NESSCA and inventory of non-ataxia signs (INAS) [6, 44]. Thus, the relationship of AOga to the speeding of progression in SCA3 is still debated. Similar to the gender effect, we speculated that it may be explained by the fact that AOga might affect the progression of non-ataxia in NESSCA and INAS but not ataxia signs in ICARS and SARA [22]. Whether there is a different influence pattern of AOga on the progression of non-ataxia or ataxia signs is worthy of further exploration. Additionally, the different time scales among studies might also help to explain these discrepancies.

Interestingly, AOga could improve the model performances and increase the effect size of CAGexp on the progression of ICARS. Similar to our study, two HD studies also used AO as a proxy for age at the time of examination and discovered consistent results. One study demonstrated that including AO increased the correlation between CAGexp and disease progression of multiple clinical rating scales by 69–159% [22]. Another study showed that CAGexp was the predictive factor of institutionalization but only after controlling for AO [23]. These findings may highlight the importance of aging process on the clinical progression of SCA3 as reported by previous studies [24, 37]. For example, the relationship between CAGexp and clinical progression could be masked by the effects of aging [37]. A significant correlation between CAGexp and progression was observed only after adjusting for age at baseline [24]. And the inconsistent results about the relationship between CAGexp repeat length and progression may also be associated with aging effects adjustment [22]. Thus, it can be inferred that the natural effects of aging associated with a later AOga adversely influenced the speed of disease progression mainly through interacting with CAGexp. The aging factors may affect functional capacity, balance abilities, motor skills, visual-perceptual abilities, and other measures typically used to quantify the severity of polyQ disease [45–50].

Our piece-wise linear growth models could be widely utilized in not only scientific research but also clinical practice. In scientific research, our predictive models can be used in experimental design studies on modifiers of progression. We can statistically correct the average effects of the major two modifiers, i.e., *ATXN3* CAGexp and AOga, thus identifying other new risk factors of progression. Besides, the predictive models are valuable for clinical trials to determine the potential efficacy of treatment. The progression prediction from our model could be employed in the baseline data analysis, essentially in the subject stratification. It is beneficial to eliminate or minimize the effects of these known predictors of progression before randomly assigning patients to different treatment branches. In clinical practice, although the current model cannot be used directly due to its not very good prediction accuracy at this stage, it can be further optimized to achieve good prediction performance with the future incorporation of more modifiers. At that time, in genetic counseling, such accurate prediction may help to assess disease progression, which is instructive for optimizing future medical plans. Meanwhile, it is also crucial for the clinical intervention by classifying low/high risk patients with predicted lower/higher progression rates or scores. Those patients in the advanced disease stage with faster progression, may also require more intensive treatment and care to delay the progression and improve their quality of life. Such individualized predictions are very useful for personalized medical management and cost-effectiveness of treatment.

This study has some limitations. We lacked other commonly used clinical assessments like SARA and objective imaging data, which prevented us from comprehensively assessing disease progression. However, the longest longitudinal study ensures better monitoring and evaluation of the trajectory of progression. We did not conduct an independent prospective study and only reanalyzed the data of Leotti et al. [18], but our novel insights could still provide new clues for future research on the progression of SCA3 or other polyQ diseases.

In the future, clinical trials in polyQ disease should ensure that different treatment groups are appropriately balanced for these factors of progression, including disease duration, CAGexp repeat length, and AOga. An imbalance may lead to the group differences in the rate of progression being unduly attributed to treatment effects. Given that AOga (a proxy for aging process) also affect the progression rate in SCA3 by interacting with CAGexp as described in HD [22, 24], we recommend to consider the effect of age or AOga to avoid spurious findings either in randomization process or in statistical analysis when examining the influences of CAGexp on progression. In fact, it may reduce sample size requirements and improve statistical power to detect treatment-induced effects. Meanwhile, future research needs to focus on the exploitation of other new modifiers to slow down the progression by targeting some new interventionable factors. As mentioned above, the acquisition and incorporation of more modifiers would contribute to further optimization of our models for more accurate predictions. Furthermore, external validations of the models in other independent SCA3 and other polyQ diseases are needed to test its adaptability, flexibility and extensibility. In addition, further transformation of our models into clinical applications will necessarily require rigorous clinical testing in large, multicenter, multi-racial/ethnic cohorts at different stages, as well as well-designed clinical assessments and neuroimaging examinations.

In conclusion, through reanalyzing the longest longitudinal study of SCA3, we offered novel insights on the disease progression using a different time scale and analysis strategies. For the first time, this present study demonstrated that the progression rate of ICARS scales was not uniform during the long duration in SCA3, varying according to the phases of disease. Different time scales may affect the results of the analysis and combining two or more times scales can provide new insights into disease progression. It has great implications for understanding the biological characteristics of disease progression in SCA3 and other polyQ diseases. In addition to *ATXN3* CAGexp repeat length, AOga or aging process may also modulate the progression in SCA3 through interacting with CAGexp. In polyQ disease, future clinical trials should take these phenomena into account, which would be conducive to determine inclusion criteria, assess treatment effect, reduce sample size requirements and increase statistical power. Moreover, our piece-wise linear growth models could facilitate genetic counseling, identification of novel modifiers on progression, personalized clinical management, and clinical trials design.

## Supplementary Information

**Additional file 1.** Parameter estimates, fit statistics of fitted models with sex or RAO as a fixed effect.

**Additional file 2.** ICARS prediction based on expanded ATXN3 CAG repeat length, AOga and duration.

**Additional file 3.** ICARS prediction based on expanded ATXN3 CAG repeat length and duration.

## Data Availability

The datasets generated during and/or analysed during the current study were publicly available at https://onlinelibrary.wiley.com/doi/10.1002/ana.25919.
