# Supplementary material for: The progression rate of spinocerebellar ataxia type 3 varies with disease stage
Source: J Transl Med. 2022 May 14;20:226. doi: 10.1186/s12967-022-03428-1 (PMC9107762; doi:10.1186/s12967-022-03428-1)
Supplement: Supplementary file 1 — Additional file 1. Parameter estimates, fit statistics of fitted models with sex or RAO as a fixed effect. [file 12967_2022_3428_MOESM1_ESM.docx]

**Additional file 1. Parameter estimates, fit statistics of fitted models with sex or RAO as a fixed effect.**

|  | **LM5** | **LM6** | **PM5** | **PM6** | **PM6c** |
| --- | --- | --- | --- | --- | --- |
| intercept | 3.007(1.930)  P=0.120 | 42.556(22.547)  P=0.060 | 41.937(2.734)  P<0.001 | -61.110(33.190)  P=0.066 | -64.290(29.809)  P=0.032 |
| dt | 3.044(0.220)  P<0.001 | -7.825(2.631)  P=0.003 | - | - | - |
| d1 | - | - | 2.783(0.264)  P<0.001 | -6.874(3.278)  P=0.036 | -7.252(2.781)  P=0.009 |
| d2 | - | - | 3.606(0.462)  P<0.001 | -7.568(6.144)  P=0.219 | -6.183(2.795)  P=0.027 |
| gender | -1.668(2.650)  P=0.531 | - | -9.919(3.770)  P=0.010 | - | - |
| dt*gender | -0.589(0.304)  P=0.053 | - | - | - | - |
| CAGexp | - | -0.592(0.332)  P=0.078 | - | 1.443(0.489)  P=0.004 | -0.362(0.297)  P=0.226 |
| dt*CAGexp | - | 0.156(0.039)  P<0.001 | - | - | 0.142(0.041)  P<0.001 |
| d1*gender |  | - | -0.657(0.366)  P=0.074 |  |  |
| d2*gender |  | - | -0.096(0.636)  P=0.881 |  |  |
| d1*CAGexp | - | - |  | 0.137(0.048)  P=0.005 | - |
| d2*CAGexp | - | - |  | 0.163(0.090)  P=0.072 | - |
| RAO | - | 0.590(0.184)  P=0.002 |  | 0.895(0.271)  P=0.002 | 0.680(0.168)  P<0.001 |
| dt*RAO | - | 0.025(0.021)  P=0.232 |  | - | 0.015(0.022)  P=0.513 |
| d1*RAO | - | - |  | 0.018(0.027)  P=0.517 | - |
| d2*RAO | - | - |  | 0.005(0.047)  P=0.917 | - |
| AIC | 4013.567 | 4005.769 | 3940.725 | 3939.014 | 3928.450 |
| BIC | 4049.133 | 4050.194 | 3998.478 | 4010.018 | 3990.623 |
| logLik | -1998.783 | -1992.884 | -1957.362 | -1953.507 | -1950.225 |
| conditional R^2^ | 0.970 | 0.972 | 0.979 | 0.980 | 0.980 |
| marginal R^2^ | 0.513 | 0.623 | 0.541 | 0.641 | 0.643 |

LM5: linear growth model (duration and gender as variables),

LM6: linear growth model (duration, CAGexp and RAO as variables),

PM5: piece-wise linear growth model (duration and gender as variables)

PM6: piece-wise linear growth model (duration, CAGexp and RAO as variables)

PM6c: piece-wise linear growth model (piece-wise fitting for duration as a variable, linear fitting for CAGexp and RAO as variables)

In this model, dt denoted the total duration of disease. Two new variables (d1 and d2) were defined for each individual: d1=dt-13 for dt≤13 and d1=0 for dt>13; while d2=0 for dt≤13, and d2 = dt-13 for dt>13.

Nakagawa R^2^, or pseudo R-squared, represents the proportion of variability in the outcome that can be explained by the fitted model. It involves two types of R^2^ (marginal and conditional R^2^). Marginal R^2^ is associated with the variance explained only by the fixed effects, while conditional R^2^ is concerned with the variance explained by the entire model (i.e., both fixed and random effects).

The value of parameter estimates of fixed effects were represented by mean [SE].

Abbreviations: SE=standard error; RAO=residual of age at onset of gait ataxia; CAGexp = expanded CAG repeat; logLike=log-Likelihood; R^2^=R-squared; ANOVA =analysis of variance.
